# Assessment of Occupational Hearing Loss Associated With Non-Gaussian Noise Using the Kurtosis-Adjusted Cumulative Noise Exposure Metric: A Cross-Sectional Survey

**DOI:** 10.3389/fpsyg.2022.870312

**Published:** 2022-04-14

**Authors:** Zhihao Shi, Xin Wang, Xiangjing Gao, Hongwei Xie, Lifang Zhou, Meibian Zhang

**Affiliations:** ^1^School of Medicine, Ningbo University, Ningbo, China; ^2^National Institute of Occupational Health and Poison Control, Chinese Center for Disease Control and Prevention, Beijing, China; ^3^Occupational Health and Radiation Protection Institute, Zhejiang Provincial Center for Disease Control and Prevention, Hangzhou, China

**Keywords:** kurtosis, non-Gaussian noise, hearing loss, cumulative noise exposure, manufacturing workers

## Abstract

**Objective:**

There is little literature on the validity of kurtosis-adjusted noise energy metrics in human studies. Therefore, this study aimed to validate the application of cumulative noise exposure (CNE) adjusted by kurtosis in evaluating occupational hearing loss associated with non-Gaussian noise among manufacturing workers.

**Methods:**

A cross-sectional survey was conducted on 1,558 manufacturing workers exposed to noise from five industries to collect noise exposure and hearing loss data. Both CNE and kurtosis-adjusted CNE (CNE′) were collapsed into 2-dB(A)∙year bins, and the mean noise-induced permanent threshold shifts at 3, 4, and 6 kHz (NIPTS_346_) in each bin were calculated. The contributions of CNE and CNE′ to noise-induced hearing loss (NIHL) were compared using the multiple linear regression. The degree of overlap of two linear regression equations (i.e., between CNE′ and NIPTS_346_ for non-Gaussian noise and between CNE and NIPTS_346_ for Gaussian noise) was used to evaluate the validity of the CNE′ using a stratified analysis based on age and sex.

**Results:**

Multiple linear regression models showed that after kurtosis adjustment, the standardized regression coefficient of CNE increased from 0.230 to 0.255, and *R*^2^ increased from 0.147 to 0.153. The linear relationship between NIPTS_346_ and CNE′ or CNE showed that the regression line of non-Gaussian noise was closer to that of Gaussian noise when using CNE′ than using CNE. The mean difference in NIPTS_346_ between the equations of non-Gaussian noise and Gaussian noise was significantly reduced from 4.32 to 1.63 dB HL after kurtosis adjustment (*t* = 12.00, *p* < 0.001). Through a stratified analysis, these significant decreases were observed in male and female workers, and workers aged ≥30 years old.

**Conclusion:**

As a noise exposure metric combining noise energy and temporal characteristics, the kurtosis-adjusted-CNE metric was more effective than CNE alone in assessing occupational hearing loss among manufacturing workers in non-Gaussian noise environment. However, more studies are needed to verify the validity of the kurtosis-adjusted-CNE metric.

## Introduction

With the development of industrialization, non-Gaussian noise has been the main noise type in the industry. Non-Gaussian noise (also known as complex noise) comprises transient high-energy impulsive noise superimposed on the steady-state background noise (Suter, 2017). Unlike steady-state noise (Gaussian noise), which has a normal or Gaussian distribution of acoustic energy in time, non-Gaussian noise has a complex distribution of acoustic energy and changes over time. Some studies found that because of complex temporal characteristics, non-Gaussian noise caused more severe hearing loss than Gaussian noise (Lei et al., 1994; Hamernik et al., 2003, 2007). This phenomenon challenges the validity of the equal energy hypothesis (EEH), which assumes that the effects of noise exposure on the cochlea are proportional to noise energy, regardless of its distribution (Suter, 2017). Since existing noise standards (e.g., ISO 1999) have been established on the basis of EEH, which use A-weighted sound pressure level (L_Aeq_) as the sole metric of noise exposure, their applicability to non-Gaussian noise is questionable. Zhang et al. (2020) found that ISO1999 underestimated noise-induced permanent threshold shift associated with non-Gaussian noise by 13.6 dB HL on average across the four audiometric test frequencies (2, 3, 4, and 6 kHz). The problem with existing noise standards is that they only rely on noise energy to quantify the noise exposure and ignore the effect of temporal characteristics on noise-induced hearing loss (NIHL). Therefore, it is necessary to develop new noise exposure metrics that can combine noise energy and temporal characteristics to effectively evaluate NIHL associated with different types of noise.

Temporal characteristics of noise waveform contain many elements such as the peak level, duration of an impulse, and inter-peak interval (Zhang et al., 2021b). Kurtosis (*β*) has been shown to incorporate these elements and can be used as a simple and feasible metric to indirectly reflect the temporal characteristics of noise (Erdreich, 1986; Hamernik and Qiu, 2001; Hamernik et al., 2003). Cumulative noise exposure (CNE) is a comprehensive metric combining noise intensity and noise exposure duration (ED), which can better represent noise energy than L_Aeq_ (Sulkowski and Lipowczan, 1982; Earshan, 1986). Studies showed that both kurtosis and CNE had a dose–response relationship with NIHL (Zhang et al., 2014; Xie et al., 2016). Thus, some scholars proposed that the CNE adjusted by kurtosis (kurtosis-adjusted CNE, CNE′) could be used as a new metric for effectively evaluating the risk of NIHL. To test this idea, Zhao et al. (2010) and Xie et al. (2016) conducted a human survey with sample sizes of 195 and 341 manufacturing workers, respectively. They took the dose–response curves between CNE (CNE′) and NIHL prevalence as an evaluation method and found that the non-Gaussian noise curve was closer to the Gaussian noise curve when using CNE′ than using CNE. However, there is still a research gap in that there are few studies on large sample sizes of workers in different industries to verify the validity of the CNE′ metric.

In this study, 1,558 manufacturing workers from five industries were included through a cross-sectional survey to test the application of CNE′ in assessing the occupational hearing loss associated with non-Gaussian noise. The contributions of CNE and CNE′ to noise-induced hearing loss (NIHL) were compared using the multiple linear regression and dose-effect curve.

## Materials and Methods

### Subjects

A cross-sectional survey was conducted from 2019 to 2021. Industrial workers exposed to noise (*N* = 2,065) were recruited from 17 manufacturing enterprises in five industries in the Zhejiang province of China. Workers from the automotive (four factories), electronics (three factories), and metal products (four factories) industries were mainly exposed to non-Gaussian noise, while those from the textile (four factories) and paper-making (two factories) industries were primarily exposed to Gaussian noise. Each participant was informed of the purpose and design of this study and was asked to sign an informed consent form. The study protocol was approved by the ethics committee of the Zhejiang Center for Disease Control and Prevention, China (approval reference number: ZJCDC-T-043-R).

The criteria for inclusion were as follows: (1) consistently working in the same job category and work site for the entire employment period; (2) being employed at their current work for at least 1 year; (3) no history of military service or shooting activities; (4) no history of ear diseases, ear trauma, or hearing loss; (5) no family history of hearing loss; (6) no history of ototoxic drug use; (7) no co-exposure to noise and ototoxic chemicals or heavy metals confirmed by field investigation; and (8) no or minimal use of hearing protection devices (HPD). As a result, 1,558 workers were included from the original pool of 2,065 participants.

### Field Investigation

Before the survey, a field investigation was conducted to understand the size and space of the workplaces, production processes, the distribution of noise resources, the noise type and noise level, the number of workers exposed to noise, and the use of HPD. The workplaces with stable work processes and machinery were selected for survey workplaces through the field investigation. Before recording, a hygienist confirmed with the manager of the workplace and each participant that this was the noise they were typically exposed to on an average working day.

### Questionnaire Survey

A face-to-face questionnaire survey was administered by an occupational hygienist. The questionnaire collected the following information from each participant: general individual information (sex, age, history of military service or shooting activities, etc.), occupational history (factory, worksite, job type, length of employment, duration of daily noise exposure, HPD use, past work with noise exposure, etc.), and health condition (history of ear diseases, ear trauma, or hearing loss, ototoxic drug use, smoking or drinking, diabetes, etc.). All information was checked for errors and then stored in the database every day.

### Noise Data Collection

A digital sound recorder (ASV5910-R, Hangzhou Aihua Instruments Co., Ltd., China) was used to record each participant’s noise exposure over the course of a shift. The instrument is a specialized device for precise measurement and analysis of personal noise exposure. It is equipped with a 1/4-inch pre-polarized condenser microphone characterized by broad frequency response (20 Hz to 20 kHz), high sensitivity level (2.24 mV/Pa), and wide measurement range (40–141 dB[A]). Under a full charge, the recorder can work continuously for at most 23 h. The full-shift noise of each participant was recorded with a 32-bit resolution at 48 kHz sampling rate. The recording was saved on 32 GB micro SD card and then transferred to a computer for subsequent analysis.

### Calculation of Noise Metrics

The MATLAB software was used to analyze the noise waveform for obtaining the kurtosis value and A-weighted sound pressure level normalized to a nominal 8-h working day (L_Aeq,8h_). A kurtosis value was computed in each consecutive 40-s time window of the noise recording. The arithmetic mean of the calculated kurtosis values in a recording was calculated and used as the kurtosis metric (*β*). Kurtosis represents the impulsiveness of noise (Qiu et al., 2021). The greater the kurtosis, the higher the impulsiveness. Kurtosis value 10 was used to distinguish non-Gaussian noise from Gaussian noise (Davis et al., 2012). Noise with kurtosis greater than or equal to 10 was defined as non-Gaussian noise, while noise with kurtosis less than 10 was defined as Gaussian noise.

L_Aeq,8h_ can be calculated by the formula in ISO 1999 2013:


(1) $$L_{\mathrm{Aeq},8h}=L_{\mathrm{Aeq},T_{e}}+10*\mathrm{lg}\left(T_{e}/T_{0}\right)$$

where *T*_e_ is the effective duration of the working day in hours; *T*_0_ is the reference duration (8 h); and L_Aeq,Te_ is the L_Aeq_ for *T*_e_. CNE, a comprehensive index combining noise intensity with exposure duration, is defined as:


(2) $$\mathrm{CNE}=10*\mathrm{lg}\left[\frac{1}{T_{\mathrm{ref}}}\overset{n}{\underset{i=1}{\sum }}\left(T_{i}*10^{L_{Aeq,8hi}/10}\right)\right]$$

where *n* is the number of stages working at different noise environments; *T*_i_ is the duration of noise exposure in years at the *i*th stage; L_Aeq,8h_*i* is the L_Aeq,8h_ occurring over the time interval *T*_i_; and T_ref_ = 1 year. Because all subjects in this study were restricted to work in the same noise environment for the entire employment period, *n* equaled to 1, and a simplified formula for Eq. (2) was given as follows:


(3) $$\mathrm{CNE}=L_{Aeq,8h}+10*lgT$$

where *T* is the duration of noise exposure. CNE′ could be used as a new metric for hearing loss risk assessment. It combines kurtosis (*β*), L_Aeq,8h_, and exposure duration (*T*), and the calculation formula is shown as follows:


(4) CNE'=LAeq,8h+lnβ+1.9lg2∗lgT

### Pure-Tone Audiometry

Each participant was given a pure-tone audiometry and an otologic examination by a certificated audiologist. The audiometric test was performed in an audiometric room of a mobile physical examination vehicle using an audiometer (Interacoustics AD629, Denmark) with an air conduction headphone (HDA300). Before the test, the audiometer and the headphone were calibrated by the Zhejiang Institute of Metrology according to the Chinese standard (Verification Regulation of Audiological Equipment Pure-tone Audiometers, JJG 388–2012).

The test was performed at least 16 h after occupational noise exposure. Air conduction pure-tone hearing threshold levels at 0.5, 1, 2, 3, 4, 6, and 8 kHz were tested in both ears. Measured hearing thresholds at each frequency were adjusted by subtracting the age- and sex-specific hearing thresholds according to Table A.3 of ISO 1999 2013. The noise-induced permanent threshold shifts (NIPTS) at each frequency for each participant were calculated according to ISO 1999 2013. The mean NIPTS at 3, 4, and 6 kHz in both ears (NIPTS_346_), representing the extent of hearing loss at high frequencies, was calculated for subsequent analysis.

### Methods for Comparing the Contribution of CNE and CNE′ to NIPTS_346_

The multiple linear regression analysis and dose-effect curve were used to compare the contribution of CNE to NIHL before and after kurtosis adjustment. In Model 1 of multiple linear regression, analysis, age, sex, and CNE were used as the independent variables, and NIPTS_346_ was used as the dependent variable. In Model 2, age, sex, and CNE′ were used as the independent variables, and NIPTS_346_ was used as the dependent variable. The standardized regression coefficient served as an indicator for comparing the contribution of CNE and CNE′ to NIPTS_346_. In addition, the value of *R*^2^, which represents the goodness-of-fit in the regression model, served as another evaluation indicator.

The dose-effect curves between CNE (CNE′) and NIPTS_346_ for Gaussian and non-Gaussian noise were plotted. Both CNE and CNE′ were collapsed into 2-dB(A)∙year bins, and the mean NIPTS_346_ in each bin was calculated. In the dose-effect curves, the abscissa was the mid-value in each bin, while the ordinate was the mean NIPTS_346_ in the corresponding bin. The differences in NIPTS_346_ between the non-Gaussian noise curve and the Gaussian noise curve at each CNE bin (D_1_) and the differences at each CNE′ bin (D_2_) were calculated and compared. Considering the influence of age and sex in NIHL, a stratified analysis is needed. Study subjects were stratified by age and sex, respectively, and then, the dose-effect curves were plotted and analyzed.

### Statistical Analysis

Continuous variables were expressed as mean with standard deviation or median with quartile. Continuous variables were compared between two groups using the *t*-test or non-parametric test. Categorical variables were expressed as proportions and were compared using the chi-square test. To compare the hearing loss caused by different noise types, an analysis of covariance was performed, in which NIPTS_346_ served as the dependent variable, noise type (non-Gaussian or Gaussian noise) served as the fixed factor, while age (≥30 years or < 30 years), sex (male or female), and CNE served as the covariates for controlling the differences in age, sex, and noise energy between two groups. The independent *t*-test was used to compare the differences between D_1_ and D_2_. *p* < 0.05 was considered significant.

## Results

### General Information of Noise Exposure

Table 1 shows the general noise exposure information for 1,558 workers in five industries. Of them, 64.4% were male. The mean age of subjects was 34.2 ± 9.3 years. The mean L_Aeq,8h_ was 89.6 ± 7.1 dB(A), and the average exposure duration was 7.3 ± 6.5 years. Among all participants, 928 workers, mainly from automotive, electronics, and metal products manufacturing industries, were exposed to non-Gaussian noise, while 630 workers, mainly from textile and paper-making industries, were exposed to Gaussian noise.

**Table 1:** The general information of noise exposure for participants from five industries.

	*N*	Male (%)	Age (year)	ED (year)	L_Aeq,8h_ [dB(A)]	CNE [dB(A)·year]	Kurtosis^*^
Automotive	589	81.3	32.6 ± 8.2	5.4 ± 4.9	87.7 ± 4.2	93.5 ± 5.6	15.0 (9.1, 25.1)
Electronics	262	47.3	31.6 ± 8.0	5.8 ± 5.2	84.6 ± 6.0	90.4 ± 7.9	24.9 (15.4, 44.0)
Metal products	194	68.0	38.3 ± 9.4	9.7 ± 8.1	91.1 ± 6.9	99.2 ± 9.2	16.0 (7.2, 48.5)
Textile	422	49.8	33.2 ± 8.5	8.6 ± 6.7	94.9 ± 7.9	102.6 ± 8.8	5.1 (3.3, 11.2)
Paper making	91	61.5	46.9 ± 9.8	11.9 ± 8.6	88.9 ± 4.5	98.2 ± 6.0	7.8 (4.8, 12.6)
Total	1,558	64.4	34.2 ± 9.3	7.3 ± 6.5	89.6 ± 7.1	96.4 ± 8.8	12.9 (6.6, 25.0)

*ED: exposure duration; CNE: cumulative noise exposure*.

* *kurtosis value was expressed as the median with quartile*.

### Comparison of NIPTS_346_ Between Non-Gaussian Noise Group and Gaussian Noise Group

The analysis of the covariance model in Table 2 shows that the least-squares means of NIPTS_346_ between non-Gaussian noise group and Gaussian noise group were 23.53 ± 0.34 dB HL (95% *CI* 22.85–24.21) and 21.53 ± 0.43 dB HL (95% *CI* 20.69–22.37), respectively. The least-squares mean difference (2.00 dB HL) of NIPTS_346_ between the two groups was significant (*p* = 0.001).

**Table 2:** Comparison of least-squares mean of NIPTS_346_ between non-Gaussian noise and Gaussian noise.

Noise type	Least-squares mean	Standard error	95% CI	*p*
Non-Gaussian noise	23.53	0.34	22.85–24.21	0.001
Gaussian noise	21.53	0.43	20.69–22.37	

### Multiple Linear Regression Analyses Between NIPTS_346_ and Key Factors

Table 3 shows the results of the multiple linear regression analyses. The two models and each factor (e.g., age, sex, CNE, and CNE′) had statistical significance (*p* < 0.001). From Model 1 to Model 2, the standardized regression coefficient of CNE increased from 0.230 to 0.255 (increased by 10.9%), while the standardized regression coefficient of age decreased from 0.231 to 0.200 (reduced by 13.4%). In Model 1, the order of the standard regression coefficient was age > CNE > sex; in Model 2, the order of the standard regression coefficient was CNE′ > age > sex. *R*^2^ increased from 0.147 for Model 1 to 0.153 for Model 2, an increase of 4.1%. *R*^2^_CNE_ and *R*^2^_CNE′_ in the non-Gaussian group were 0.732 and 0.770, respectively, an increase of 5.2%.

**Table 3:** The multiple linear regression analyses between NIPTS_346_ and key factors.

	Unstandardized coefficient	Standardized coefficient	*t*	*p*
Model 1: NIPTS_346_ = b_0_ + b_1_Age + b_2_Sex + b_3_CNE			*R*^2^_model 1_ = 0.147	*R*^2^_CNE_ = 0.732^*^
Intercept (b_0_)	−20.462		−4.965	*p* < 0.001
Age (b_1_)	0.271	0.231	6.852	*p* < 0.001
Sex (b_2_)	1.849	0.081	2.588	*p* < 0.001
CNE (b_3_)	0.326	0.230	6.965	*p* < 0.001
Model 2: NIPTS_346_ = b_0_ + b_1_Age + b_2_Sex + b_3_CNE′			*R*^2^_model 2_ = 0.153	*R*^2^_CNE’_ = 0.770^+^
Intercept (b_0_)	−16.968		−4.818	*p* < 0.001
Age (b_1_)	0.234	0.200	5.687	*p* < 0.001
Sex (b_2_)	2.008	0.088	2.835	*p* < 0.001
CNE′ (b_3_)	0.286	0.255	7.405	*p* < 0.001

* *R^2^_CNE_ was the R^2^ for CNE in the linear regression model between mean NIPTS_346_ and CNE (collapsed into 2-dB(A)∙year bins) in the non-Gaussian noise group*.

+ *R^2^_CNE’_ was the R^2^ for CNE′ in the linear regression model between mean NIPTS_346_ and CNE′ (collapsed into 2-dB(A)∙year bins) in the non-Gaussian noise group*.

### The Dose-Effect Relationships Between NIPTS_346_ and CNE or CNE′

**Figure 1:**
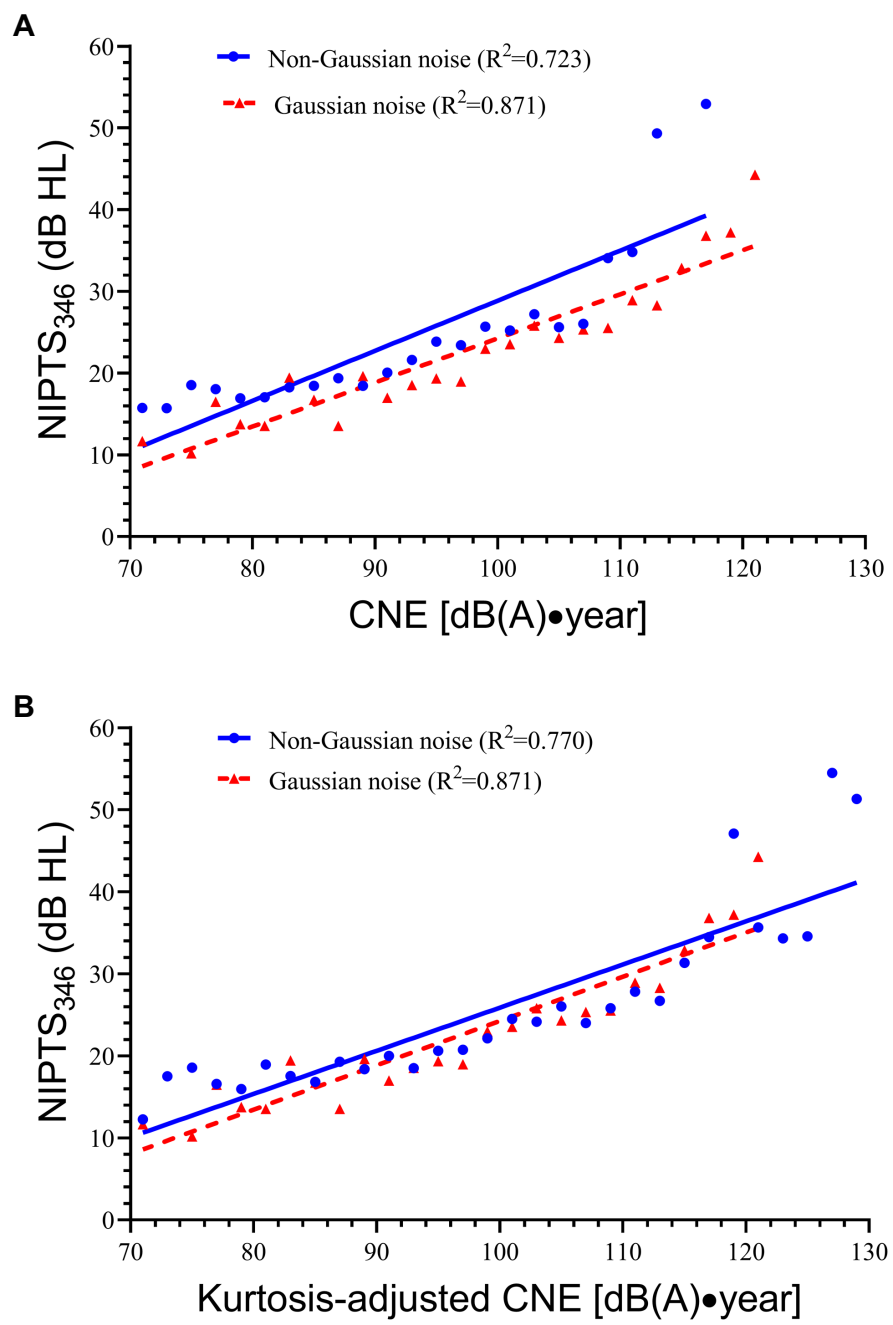
The linear relationship between NIPTS_346_ and CNE or CNE′ for all subjects. **(A)** The linear relationship between NIPTS_346_ and CNE. The regression equation for Gaussian noise is NIPTS_346_ = 0.540CNE—29.707, *R*^2^ = 0.871. The regression equation for non-Gaussian noise is NIPTS_346_ = 0.613CNE′—32.415, *R*^2^ = 0.723. **(B)** The linear relationship between NIPTS_346_ and CNE′. The regression equation for non-Gaussian noise is NIPTS_346_ = 0.526CNE′—26.697, *R*^2^ = 0.770.

The simple linear regression model was used to fit the dose-effect curve between NIPTS_346_ and CNE or CNE′. Figure 1A demonstrates the linear regression equation between NIPTS_346_ and CNE for both the non-Gaussian noise group and the Gaussian noise group. The simple linear regression equation of the Gaussian noise group was NIPTS_346_ = 0.540CNE—29.707, *R*^2^ = 0.871. The equation of non-Gaussian noise group was NIPTS_346_ = 0.613CNE′—32.415, *R*^2^ = 0.723. The regression line of non-Gaussian noise (continuous line) was above the line of Gaussian noise (dotted line) with a significant distance between them. Figure 1B shows the linear relationship between NIPTS_346_ and CNE′. The equation of the Gaussian noise group remained unchanged, while that of the non-Gaussian noise group was changed to NIPTS_346_ = 0.526CNE′—26.697, *R*^2^ = 0.770. After CNE was adjusted by kurtosis, the regression line of non-Gaussian noise was closer to that of Gaussian noise, and *R*^2^ of non-Gaussian noise had an increase of 6.5% (from 0.723 to 0.770). Table 4 shows the mean difference in NIPTS_346_ between the non-Gaussian noise equation and the Gaussian noise equation at each bin before and after the kurtosis adjustment. The two independent samples *t*-test showed that the mean D_2_ of NIPTS_346_ was 1.63 dB HL, which was significantly lower than D_1_ (4.32 dB HL; *t* = 12.00, *p* < 0.001).

**Table 4:** A decrease in NIPTS_346_ difference between the two equations of non-Gaussian and Gaussian noise after the kurtosis adjustment.

Factor	Mean D_1_ (dB HL)	Mean D_2_ (dB HL)	*t*	*p*
Total	4.32	1.63	12.00	<0.001
Male	3.47	0.96	20.11	<0.001
Female	5.26	2.04	14.25	<0.001
Age ≥ 30	4.10	1.13	15.80	<0.001
Age < 30	2.70	2.53	0.38	0.707

*D_1_, the difference between the two linear regression equations of non-Gaussian and Gaussian noise at each unadjusted CNE bin*.

*D_2_, the difference between the two linear regression equations of non-Gaussian and Gaussian noise at each CNE′ bin*.

**Figure 2:**
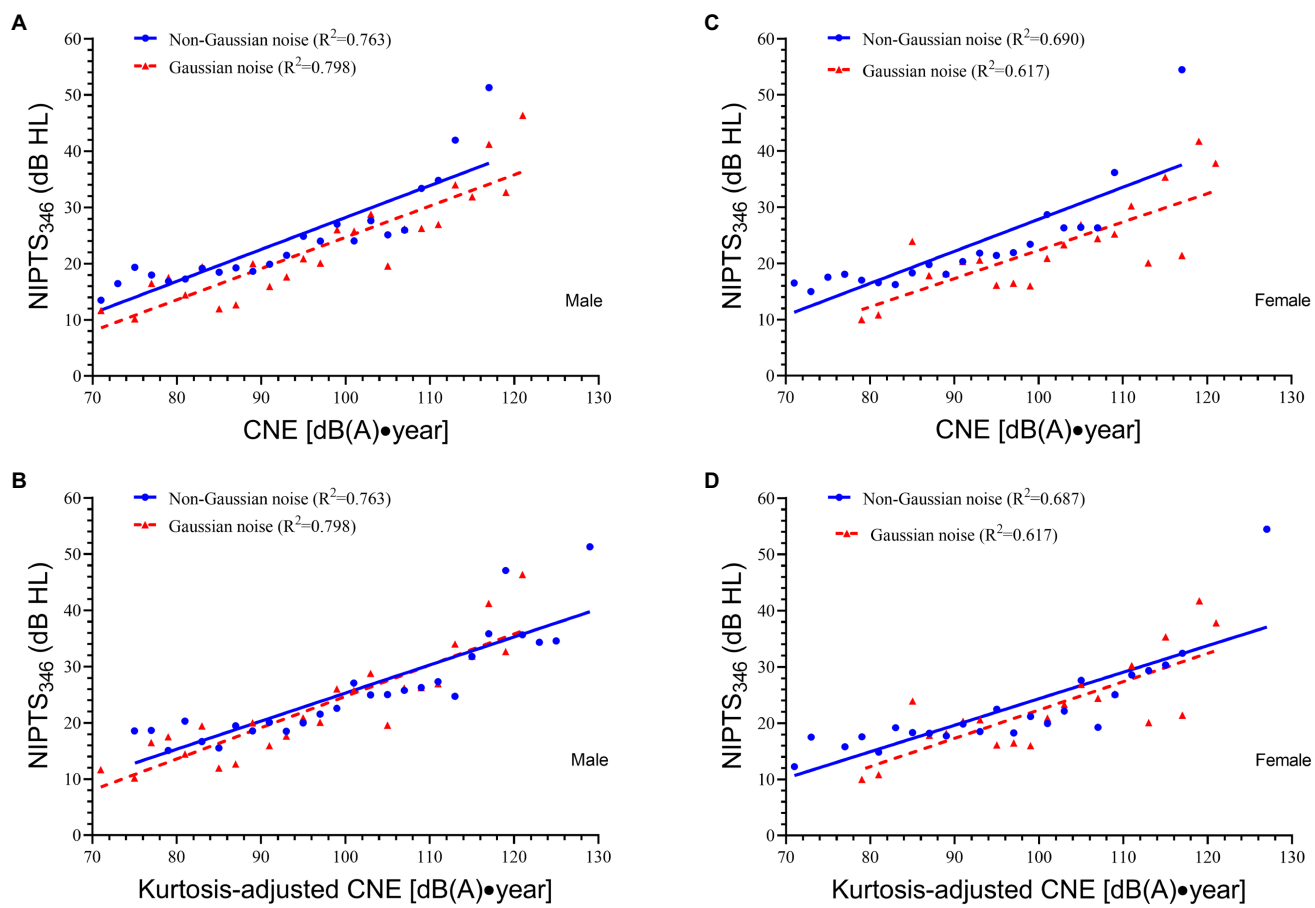
The linear relationship between NIPTS_346_ and CNE or CNE′ for male and female workers. **(A)** The linear relationship between NIPTS_346_ and CNE for male workers. The regression equation for Gaussian noise is NIPTS_346_ = 0.556CNE—30.910, *R*^2^ = 0.798. The regression equation for non-Gaussian noise is NIPTS_346_ = 0.568CNE′—28.599, *R*^2^ = 0.763. **(B)** The linear relationship between NIPTS_346_ and CNE′ for male workers. The regression equation for non-Gaussian noise is NIPTS_346_ = 0.499CNE′—24.598, *R*^2^ = 0.763. **(C)** The linear relationship between NIPTS_346_ and CNE for female workers. The regression equation for Gaussian noise is NIPTS_346_ = 0.504CNE—28.037, *R*^2^ = 0.617. The regression equation for non-Gaussian noise is NIPTS_346_ = 0.571CNE′—29.231, *R*^2^ = 0.690. **(D)** The linear relationship between NIPTS_346_ and CNE′ for female workers. The regression equation for non-Gaussian noise is NIPTS_346_ = 0.472CNE′—22.825, *R*^2^ = 0.687.

Figures 2A,B show the linear regression equations for male workers when using both CNE and CNE′, and Figures 2C,D for female workers. When using CNE, the regression line of non-Gaussian noise for both males and females was above that of Gaussian noise with a significant distance between them (male: mean *D*_1_ = 3.47 dB HL; female: mean *D*_1_ = 5.26 dB HL). When CNE′ was used, the regression line of non-Gaussian noise for males nearly overlapped with that of Gaussian noise (mean *D*_2_ = 0.96 dB HL). For females, the regression line of non-Gaussian noise was also very close to that of Gaussian noise (mean *D*_2_ = 2.04 dB HL). The mean difference when using CNE (*D*_1_) was significantly higher than that when using CNE′ (*D*_2_) for both males (*t* = 20.11, *p* < 0.001) and females (*t* = 14.25, *p* < 0.001).

**Figure 3:**
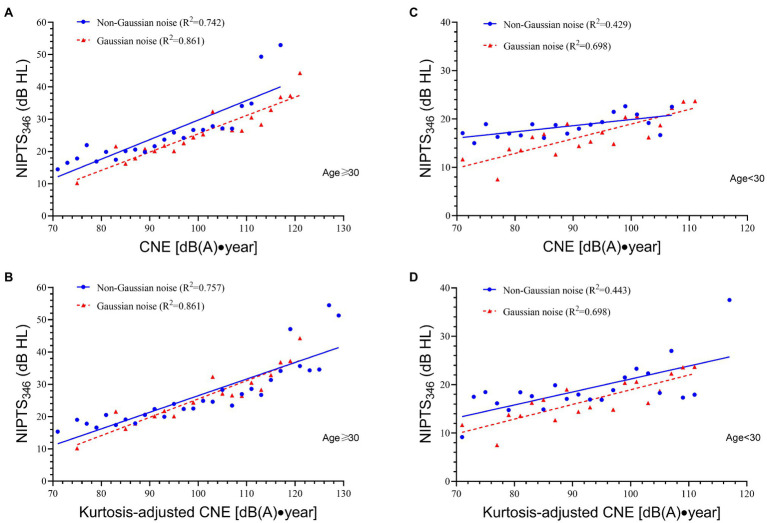
The linear relationship between NIPTS_346_ and CNE or CNE′ for workers aged ≥30 and aged <30. **(A)** The linear relationship between NIPTS_346_ and CNE for workers aged ≥30. The regression equation for Gaussian noise is NIPTS_346_ = 0.567CNE—31.269, *R*^2^ = 0.861. The regression equation for non-Gaussian noise is NIPTS_346_ = 0.606CNE′—30.916, *R*^2^ = 0.742. **(B)** The linear relationship between NIPTS_346_ and CNE′ for workers aged ≥30. The regression equation for non-Gaussian noise is NIPTS_346_ = 0.514CNE′—24.925, *R*^2^ = 0.757. **(C)** The linear relationship between NIPTS_346_ and CNE for workers aged <30. The regression equation for Gaussian noise is NIPTS_346_ = 0.304CNE—11.397, *R*^2^ = 0.698. The regression equation for non-Gaussian noise is NIPTS_346_ = 0.128CNE′—7.132, *R*^2^ = 0.429. **(D)** The linear relationship between NIPTS_346_ and CNE′ for workers aged <30. The regression equation for non-Gaussian noise is NIPTS_346_ = 0.268CNE′—5.640, *R*^2^ = 0.443.

Figures 3A,B show the regression lines for workers aged 30 years or older, and Figures 3C,D for workers less than 30 years old. For workers aged ≥30, the line of non-Gaussian noise was above that of Gaussian noise when using CNE and became close to the line of Gaussian noise when using CNE′. The mean difference of NIPTS_346_ between two lines significantly decreased after CNE was adjusted by kurtosis (mean *D*_1_ = 4.10 dB HL, mean *D*_2_ = 1.13 dB HL, *t* = 15.80, *p* < 0.001). For workers aged <30, the mean difference of NIPTS_346_ when using CNE (mean *D*_1_ = 2.70 dB HL) was a little higher than CNE′ (mean *D*_2_ = 2.53 dB HL), although the difference was not statistically significant (*t* = 0.38, *p* = 0.707).

## Discussion

An analysis of covariance showed that the least-squares mean of NIPTS_346_ in the non-Gaussian group was significantly higher than that in the Gaussian noise group (*p* = 0.001), indicating that non-Gaussian noise resulted in more hearing loss than Gaussian noise under the same noise energy exposure. Other studies reported similar results. Li et al. (2021) compared the difference of hearing loss between general machinery manufacturing workers exposed to non-Gaussian noise and workers exposed to Gaussian noise (such as spinning and weaving) and found that the former had a higher threshold level of hearing. Xie et al. (2021) reported that workers in industries with high kurtosis (such as furniture, hardware, automotive, machinery, steel, and electrical equipment manufacturing industries) suffered from more severe hearing loss than workers in industries with low kurtosis values. Shi et al. (2021) conducted a meta-analysis on 30 studies covering a wide range of industries and found that workers exposed to non-Gaussian noise had 2.2 times higher risk of high-frequency NIHL than those exposed to Gaussian noise.

The increased risk of hearing loss may be associated with the complex temporal structure of non-Gaussian noise. The degree to which noise intensity deviates from Gaussian distribution (i.e., the impulsiveness of noise) is responsible for excessive hearing loss. Kurtosis is a statistics metric of the extent to which the tails of distribution differ from the tails of the Gaussian distribution. The more impulsive the noise, the greater the kurtosis. Zhang et al. (2021b) reported that kurtosis was significantly associated with the difference of peak SPL (L_peak_) minus its L_Aeq,8h_ across different types of work. The temporal structure of a non-Gaussian noise can be indirectly characterized by estimating the kurtosis. Qiu and his colleagues exposed chinchillas to noise with different kurtosis but equal energy and found that noise with higher kurtosis caused more severe hair cell loss (Qiu et al., 2006, 2007, 2013). Zhang et al. (2021a) found besides L_Aeq,8h_ and exposure duration, kurtosis was a risk factor for occupational NIHL and had a dose-effect relationship with NIPTS_346_. These findings suggest that noise energy is a necessary metric while kurtosis is also an important metric in assessing the hearing loss associated with non-Gaussian noise, and solely noise energy metrics may underestimate the hearing loss caused by non-Gaussian noise (Suvorov et al., 2001; Seixas et al., 2012; Zhang et al., 2020). Qiu et al. (2013) found that different temporal structure of noises might produce the same kurtosis value; however, for the same kurtosis, the detailed temporal structure of noise exposure did not have a strong influence on hearing trauma, while different kurtosis levels had significant influence on hearing trauma. Therefore, kurtosis and energy are sufficient and necessary metrics to evaluate NIHL. A combination of noise energy and kurtosis (e.g., kurtosis-adjusted CNE) has the potential to be used to evaluate the hearing loss associated with non-Gaussian noise.

This study aimed to validate the applicability of kurtosis-adjusted CNE (CNE′) in assessing NIHL. Multiple linear regression models in Table 3 showed the most significant standard regression coefficient in Model 1 was age, while the largest one in Model 2 was CNE′. From Model 1 to Model 2, the impact of age on NIHL decreased while the impact of CNE and kurtosis increased, indicating that kurtosis adjustment made the contribution of CNE′ to NIHL greater than that of CNE. An increase of *R*^2^ after kurtosis adjustment implied an improvement in regression goodness-of-fit, suggesting that CNE′ was a better measure for assessing NIHL associated with non-Gaussian noise than CNE. This result was supported by a study by Xie et al. (2016) that reported an increase of *R*^2^ of CNE after kurtosis adjustment using the multiple regression analysis. The larger sample size in this study (928 non-Gaussian-exposed workers) might be more convincing in terms of the validity of CNE′ than that (178 non-Gaussian-exposed workers) in Xie et al.’s study.

Figure 1 illustrates the linear relationship between NIPTS_346_ and CNE or CNE′ for all subjects. Before the kurtosis adjustment, the regression equation of non-Gaussian noise had higher levels of NIPTS_346_ than that of Gaussian noise (mean *D*_1_ = 4.32 dB HL), which was consistent with the above finding that non-Gaussian noise caused more severe hearing loss than Gaussian noise. Thus, as shown in Figure 1A, the regression line of non-Gaussian noise was above that of Gaussian noise. However, after CNE was adjusted by kurtosis, the difference of NIPTS_346_ between the two lines was significantly reduced, and the regression line of non-Gaussian noise nearly overlapped that of Gaussian noise when using CNE′ (1.63 dB HL left), which indicated that there was an equivalent noise-induced effect for the two groups. This result suggested CNE′ could be used to evaluate the hearing loss caused by different types of noise (e.g., Gaussian and non-Gaussian noise). Zhao et al. (2010) and Xie et al. (2016) came to similar conclusions. They plotted the dose–response curve between CNE (CNE′) and NIHL prevalence and found that the curve of non-Gaussian noise almost overlapped that of Gaussian noise when using CNE′. Zhang et al. (2021b) also plotted the dose–response curves and further calculated the differences in NIHL prevalence between the non-Gaussian noise group and Gaussian noise group; the authors found that after kurtosis adjustment, the average difference of NIHL prevalence significantly decreased from 7.63% to 1.12%. These findings suggested that CNE′ was able to consistently estimate the prevalence of hearing loss across varied noise environments using a single metric.

In this study, the multiple regression analysis demonstrated age and sex were risk factors affecting NIHL. This result was supported by previous studies (Gates et al., 1990; Pearson et al., 1995; Sriopas et al., 2017; Nyarubeli et al., 2019). Thus, this study used a stratified analysis based on age and sex to observe the role of CNE′ alone in NIHL. Figure 2 illustrated that in male or female workers, the use of CNE′ could significantly reduce the difference of hearing loss between non-Gaussian noise and Gaussian noise (*p* < 0.001). Especially for male workers, the regression line of non-Gaussian noise nearly overlapped that of Gaussian noise (mean *D*_2_ = 0.96 dB HL). Xie et al. (2016) also conducted a stratified analysis and obtained the same conclusion among male workers. Figures 3A,B demonstrated in workers aged ≥30, the regression line of non-Gaussian noise nearly overlapped that of Gaussian noise (mean *D*_2_ = 1.13 dB HL), and the distance between two lines was significantly reduced (*t* = 15.80, *p* < 0.001) after kurtosis adjustment.

In this study, the effectiveness of CNE′ among workers aged <30 was not significant, which was a limitation for this study. The reason was related to the insufficient sample size of these young workers in specific bins of CNE (CNE′), especially in 70–78 CNE (CNE′) bins, which increased the variability of data and resulted in low *R*^2^ values (e.g., 0.429–0.698) of regression lines. For example, for Gaussian-exposed workers, the sample size of the 70–72 CNE bin or the 76–78 CNE bin was only one and that of the 72–74 and 74–76 CNE bin was zero. Therefore, greater sample sizes of young workers exposed to low noise level are needed in further studies. In addition, methodologies to verify the effectiveness of CNE need to be further improved.

## Conclusion

As a noise exposure metric combining noise energy and temporal characteristics, the kurtosis-adjusted-CNE metric was more effective than CNE alone in assessing NIHL among manufacturing workers in the non-Gaussian noise environment. More epidemiological studies are needed to verify the validity of the kurtosis-adjusted-CNE metric.

## Data Availability Statement

The raw data supporting the conclusions of this article will be made available by the authors, without undue reservation.

## Ethics Statement

The studies involving human participants were reviewed and approved by the Zhejiang Center for Disease Control and Prevention, China (approval reference number: ZJCDC-T-043-R). The patients/participants provided their written informed consent to participate in this study.

## Author Contributions

ZS: investigation, formal analysis, and writing—original draft. XW and HX: methodology and investigation. XG: investigation and data curation. LZ: formal analysis and visualization. MZ: conceptualization, funding acquisition, writing—review and editing, and supervision. All authors contributed to the article and approved the submitted version.

## Funding

This research was funded by the Zhejiang Provincial Key Research and Development Project (grant number: 2015C03039); the Zhejiang Provincial Program for the Cultivation of High-Level Innovative Health Talents, Zhejiang Province, China; the Pre-research Project on Occupational Health Standards (20210102); and the Health Commission of Zhejiang Province (grant numbers: 2019KY057 and 2021KY120).

## Conflict of Interest

The authors declare that the research was conducted in the absence of any commercial or financial relationships that could be construed as a potential conflict of interest.

## Publisher’s Note

All claims expressed in this article are solely those of the authors and do not necessarily represent those of their affiliated organizations, or those of the publisher, the editors and the reviewers. Any product that may be evaluated in this article, or claim that may be made by its manufacturer, is not guaranteed or endorsed by the publisher.
